# UV spectrophotometric methods for simultaneous determination of ketorolac tromethamine and olopatadine hydrochloride: Application of multiple standard addition for assay of ophthalmic solution

**DOI:** 10.1038/s41598-023-45378-8

**Published:** 2023-10-24

**Authors:** Sherin F. Hammad, Mona M. Rady, Samah F. El-Malla

**Affiliations:** Department of Pharmaceutical Analytical Chemistry, Faculty of Pharmacy, Medical Campus of Tanta University, Elgeish Street, Tanta, 31111 Egypt

**Keywords:** Analytical chemistry, Diseases

## Abstract

Ophthalmic preparations that contain ketorolac tromethamine (KET) and olopatadine HCl (OLO) are used to relieve seasonal allergies and allergic conjunctivitis. Simultaneous quantification of KET and OLO was held by validated and simple spectrophotometric methods. KET was determined directly from the fundamental UV absorption spectra (at 323 nm), while OLO was determined after performing either dual wavelength or ratio derivative methods. The first method was based on measuring the absorbance difference (ΔA) between 243 and 291 nm, while the second depended on generating first derivative ratio spectra using 3.0 µg/mL KET as a divisor and measuring OLO responses at 234 nm (minima). Multiple standard addition method was applied to enable the determination of OLO which is considered as the weakly absorbing species as well as the minor component in a challenging dosage form ratio (4:1). The linearity ranges of the developed methods were 3–12 μg/mL and 4–40 μg/mL for KET and OLO, respectively. Simultaneous determination of both drugs was successfully implemented to lab prepared eye drops that contain KET, OLO and benzalkonium chloride as an inactive ingredient. Greenness assessment indicates minimal impact on environment. The developed methods determined the cited drugs with % recovery ± SD of 99.63 ± 0.01 for KET, 100.90 ± 0.02 and 100.31 ± 0.01 for OLO using dual wavelength and first derivative ratio methods, respectively. Using F-test and t-test at confidence level %95 to compare between the results of the presented methods and a reported method show no significant difference which allows precise, accurate, rapid, and simple quantification of quality control samples that contain KET and OLO.

## Introduction

**Figure 1:**
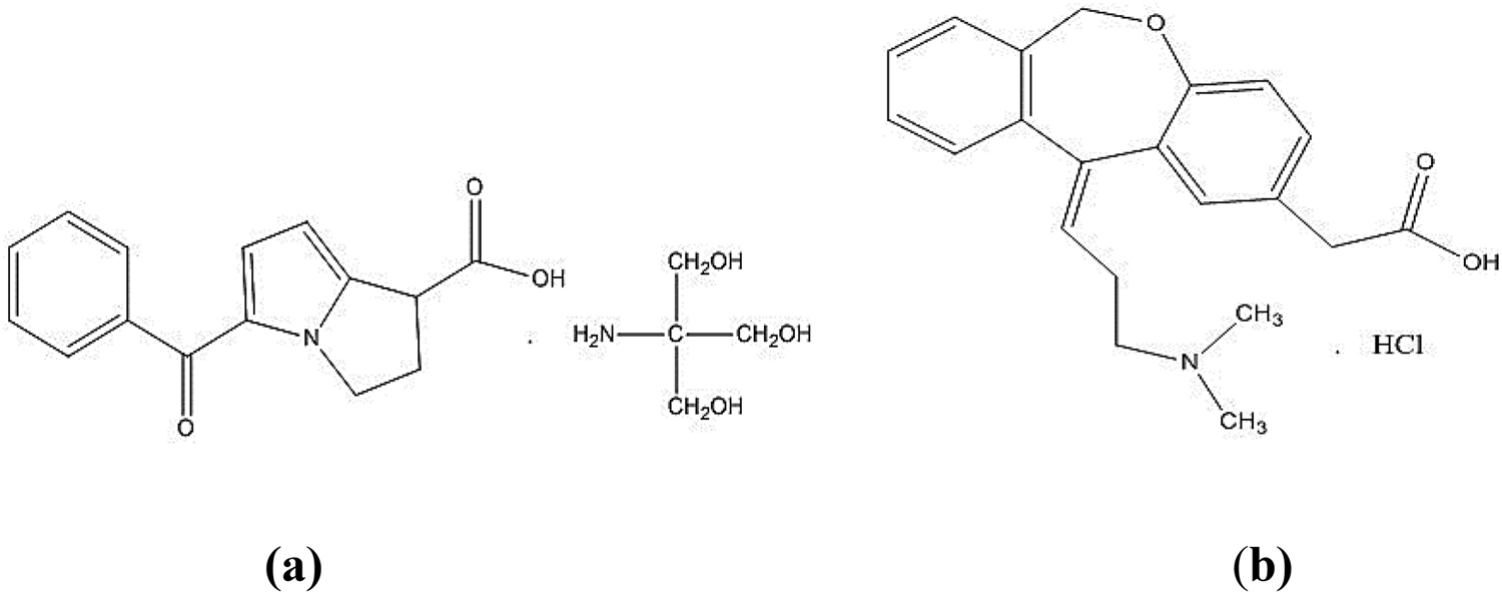
Chemical structures of KET (**a**) and OLO (**b**).

Ketorolac tromethamine (KET) (Fig. 1a) is a non-steroidal anti-inflammatory drug with analgesic, anti-inflammatory and anti-pyretic effects. It is chemically designated as 5-benzoyl-2,3 dihydro-1H-pyrrolizine-1-carboxylic acid,2-amino-2-(hydroxy methyl)-1,3-propanediol. KET is a white crystalline powder, its freely soluble in water, with pKa of 3.5, and melting point range of 162–165 °C ^1^. Olopatadine hydrochloride (OLO) (Fig. 1b) exerts its action by two mechanisms, selective H_1_-receptor antagonism and mast cell stabilization effect ^2^. The chemical name of OLO is {(11Z)-11-[3-(dimethylamino) propylidene]-6,11-dihydrodibenzo[b,e] oxepin-2-yl}acetic acid. It is a white crystalline powder, freely soluble in water. Its pKa is 3.78, and 9.76. The melting point for OLO is 248 °C ^1^. Fixed dose combination (FDC) containing KET and OLO is used to relieve eye itching and discomfort caused by seasonal allergies and allergic conjunctivitis ^3^. It contains both drugs in a ratio of 4:1 (KET: OLO), where OLO is both the minor component and that of lower absorptivity. This constitutes a challenging issue when determining OLO in the existence of strongly absorptive KET in the ratio of dosage form. This challenge is one of the most important issues targeted by much scientific researches in the field of drug analysis^4,5^.

Reviewing literature reveals various reported methods for quantification of KET either alone or in combinations, e.g., UV spectrophotometry ^6–8^, HPTLC ^9^ and HPLC ^10–12^. Determination of OLO were performed by diverse analytical methods which were reviewed by Mahmoud *et al.* ^13^. Other methods include UV spectrophotometry ^14^ and liquid chromatography ^15,16^. Simultaneous determination of both drugs were done by limited reported analytical methods such as: HPLC ^17^ and UV spectrophotometry ^18,19^.

The development and validation of two simple spectrophotometric methods for simultaneous determination of KET and OLO taking into consideration the challenging dosage form ratio and the existence of benzalkonium chloride as UV absorbing inactive ingredient that may interfere with the determination is the aim of this work. These methods were dual wavelength method (DWM) and first derivative ratio method (^1^D_R_). The developed methods did not need prior separation or expensive/toxic solvents, sophisticated instruments, or complicated procedures. Moreover, they are simple, ecofriendly, and have higher sensitivity than another reported spectrophotometric methods.

## Materials

### Instrument

UV/Vis double beam spectrophotometer, Shimazu, model 1800 (Kyoto, Japan) that is equipped with 1-cm quartz cells. Spectra recording and carrying out diverse mathematical manipulations were done using UV 2.33 probe software.

### Chemicals

PHARCO Pharmaceuticals (Alexandria, Egypt) is the supplier of KET (98.81% ± 0.05). OLO (99.20% ± 0.08) was provided from EPICO pharmaceuticals (Cairo, Egypt). Purity for both drugs was calculated by applying reported method. Sigma for Pharmaceutical Industries Co. (Quesna; Menoufia; Egypt) kindly donated benzalkonium chloride (50% w/w).

### Standard solutions

A stock standard solution containing 1 mg/mL of KET and OLO were prepared separately in distilled water in 25-mL volumetric flasks. A working standard solution containing 20.0 µg/mL KET was prepared by diluting 2.0 mL of the previous stock standard solution to 100 mL using distilled water. A working standard solution containing 50.0 µg/mL OLO was prepared by diluting 5.0 mL of the previous OLO stock standard solution to 100 mL with distilled water. The prepared stock standard solutions were stable when kept at 4 °C for 1 month.

### Assay methods and construction of calibration curves

#### Determination of KET

Calibration standards in the range of 3.0–12.0 µg/mL in water were prepared using KET working standard solution. The UV spectra of KET solutions were recorded in the range of 200–400 nm (scanning speed: fast, sampling interval: 1 nm), smoothed at dλ 1 nm and saved in the computer. The absorbance of KET was recorded at λ_max_ 323 nm directly from zero order spectra. Plotting A_323nm_ versus the concentration in µg/mL is used for construction of calibration curve and computation of regression equation.

#### Determination of OLO

OLO working standard solution was used to prepare calibration standards in the range of 4.0–40.0 µg/mL in water. The range of 200–400 nm was selected to record the UV spectra of OLO solutions. Determination of OLO were established by two methods.

##### a. Dual wavelength method (DWM)

Using the smoothed fundamental ^0^D spectra of OLO, measurements were performed at two wavelengths: 243 nm and 291 nm and, the absorbance difference (ΔA) was calculated. The calibration curve was generated by plotting ΔA_243-291nm_ against OLO concentration (µg/mL) and regression equation was estimated.

##### b. First derivative ratio spectrophotometry (^1^D_R_)

The UV spectra of OLO were divided by the smoothed ^0^D-spectrum of 3.0 µg/mL KET to get the ratio spectra. Then the first derivative ratio (^1^D_R_) spectra were generated at delta lambda 1 nm and scaling factor of 1 nm. OLO responses were measured at 234 nm (minima). Plotting the amplitudes ^1^D_R_ at 234 nm against concentration (µg/mL) were used for constructing calibration curve and then the regression equation was derived.

Three Lab-prepared binary mixtures (4, 6, 6 and 40, 20, 10 µg/mL of KET and OLO, respectively) were prepared to maintain the accuracy and precision of the established methods.

### Application to Lab-prepared eye drops

Eye drops that contain KET/OLO combination is not available in Egypt. The dosage form is composed of 0.4% w/v KET, 0.1% w/v OLO and, 0.01% w/v benzalkonium chloride. The mixture was prepared in lab to simulate the dosage form by mixing 10 mg of OLO, 40 mg of KET and 1 mg benzalkonium chloride. The mixture was dissolved in 10 mL distilled water. The prepared stock test solution contains 1 mg/mL OLO, 4 mg/mL KET, 0.1 mg/mL benzalkonium chloride. An aliquot of 2.0 mL from the previous stock solution was diluted to 100 mL in water to prepare a working assay solution containing 20.0 µg/mL OLO, 80.0 µg/mL KET and 2.0 µg/mL benzalkonium chloride. Determination of KET in lab-prepared eye drops was performed by diluting 1.0 mL of the working assay solution to 10 mL using distilled water (n = 3). Concentration was determined directly from the regression equation derived for KET determination as mentioned in section "Determination of KET" and the mean value of % recovery and % R.S.D were calculated.

For determination of OLO, different volumes of 50.0 µg/mL OLO working standard solution (2, 3, 4, 5, 6 and 7 mL) were transferred to a series 10-mL volumetric flasks each containing 1.0 mL of the previously mentioned working assay solution and completed to 10-mL with D.W. Each assay solution is prepared in triplicate. Standard addition curves were established (n = 3) by plotting the response measured (ΔA_243-291nm_ for DWM and ^1^D_R-234 nm_ for ratio spectroscopy) versus concentration added of OLO standard (μg/mL) and the x-intercept of the standard addition line was used to calculate the concentration of OLO in eye drops and the mean value of % recovery and % R.S.D were calculated ^20^.

## Results and discussion

UV spectrophotometry is the simplest, and the often-used technique in drug analysis. Most active ingredients used in formulation of drug products contain chromophores that could absorb UV radiation, making UV spectrophotometry a reliable and simple tool for their determination. Merging UV spectrophotometric measurements with chemometrics enables the continuous development of new methods for solving different analytical challenges. UV spectrophotometric technique offers simple, cost effective and rapid alternative to chromatographic techniques^21^. The presence of conjugated chromophores in OLO and KET makes UV spectrophotometry a good choice to apply for their determination. Few UV spectrophotometric methods were reported for the simultaneous determination of OLO and KET ^18,19^.

**Figure 2:**
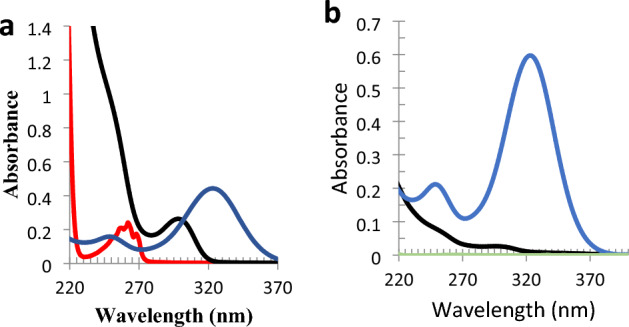
Zero-order UV absorption spectra of (**a**) KET (blue) (12 μg/mL), OLO (black) (40 μg/mL), benzalkonium chloride (red) (250 μg/mL), and (**b**) KET (blue) (8 μg/mL) and OLO (black) (2 μg/mL) and benzalkonium chloride (green) (0.2 μg/mL) in the dosage form ratio.

However, both drugs have moderately overlapped spectra and the use of benzalkonium chloride as a preservative (inactive substance) may interfere in their determination (Fig. 2a). Overlay UV spectra of solutions in the ratio of the dosage form (Fig. 2b) shows that no interference could be expected from benzalkonium chloride due to both low absorptivity at 254 nm induced by the benzenoid chromophore and very low concentration of the preservative in the dosage form. Moreover, the extended UV absorption spectrum of KET enables its easy and direct determination at 323 nm (λ_max_ of KET), however OLO determination is challenging. Not only KET shows strong absorbance at λ_max_ of OLO (292 nm), but also its UV spectrum revealed very high absorptivity across all the UV wavelength range (200–400 nm). Mathematically assisted spectrophotometric techniques like DWM and ^1^D_R_ are expected to face challenges concerning the determination of OLO in presence of KET.

### Theory of the developed UV spectrophotometric methods

#### Dual wavelength method (DWM)

**Figure 3:**
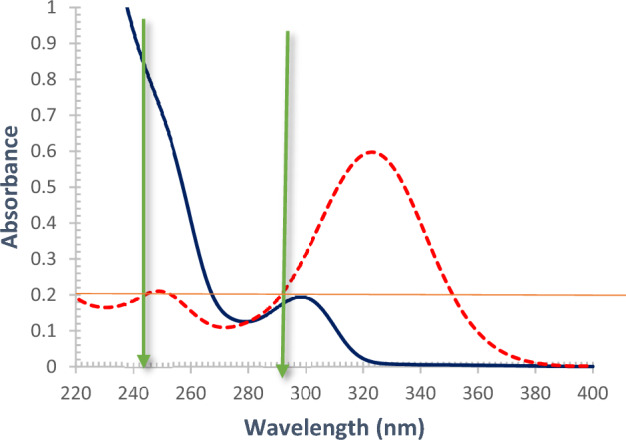
Overlay UV spectra showing selected wavelengths for determination of OLO using DWM. Concentration of KET (red) is 8 µg/mL and OLO (blue) is 30 µg/mL. Green arrows indicate the two selected wavelengths.

The principle of DWM depends on measurement of absorbance difference (ΔA) at two selected wavelengths where KET is considered as an interferent and thus its absorbance values should be equal at the selected wavelengths. The absorbance difference at 243 nm and 291 nm is directly proportional to OLO concentration while independent on that of KET (Fig. 3) ^22^.

#### First derivative ratio spectrophotometry (^1^D_R_)

**Figure 4:**
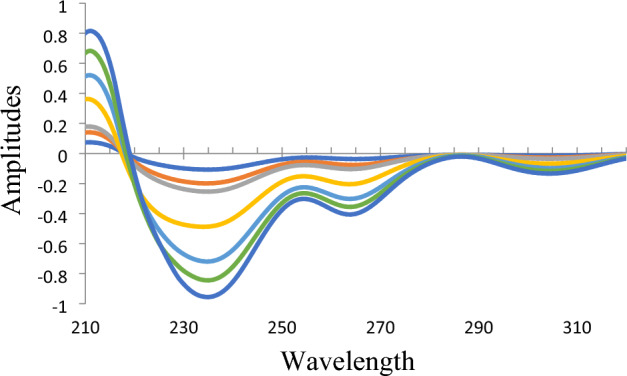
Ratio derivative spectra for OLO (at concentrations: 4–40 µg/mL) after using 3.0 µg/mL KET as a divisor and performing first derivative Δ λ = 1.

The principle of ^1^D_R_ method relies on the derivation of the ratio spectra for analyzing binary mixtures ^23^. For establishing this method, OLO is considered as the constituent of interest which is needed to be quantified in the presence of KET as an interfering constituent that is selected as a divisor. ^1^D_R_ method was performed in two steps manner. Selection of the best divisor concentration was performed first. Various concentrations of KET were tried as divisors, and then the intensity and shape of derivative ratio peaks were examined. KET with concentration 3 μg/mL was selected as the optimum divisor as it gives the highest intense, sharp, and smoothed peaks. Then performing differentiation at different orders (1st, 2nd, 3rd, and 4th derivative) of ratio spectra at different applied Δλ (1, 2, 4, or 8) to achieve optimum ratio derivative peaks with the highest amplitude, the least noisy spectrum without losing any significant spectral data. First derivative spectra at Δλ = 1 was preferred as the optimum differentiation order. Wavelength of 234 nm (minima) was selected for quantification of OLO as the highest optima that allows more method’s sensitivity (Fig. 4).

### Method validation

ICH guidelines Q2R1 ^24^ was revised to ensure methods validation. The resulting validation parameters were handled.

#### Linearity

The linearity of the established methods was maintained for KET and OLO by plotting the concentrations of KET against absorbance measured at 323 nm (***A***_323 ***nm***_). For OLO, concentration is plotted against the amplitude (^1^D_R-234nm_) in 1st derivative of ratio spectra and absorbance difference (ΔA_243-291nm_) in DWM. With aid of Microsoft Excel, regression analysis was achieved and different analytical parameters for determination of KET and OLO were calculated as shown in Table 1. Standard deviations around intercept “Sa” with small value, correlation coefficient (r) with high value, slope “S_b_” and the residuals “S_y/x_” signify the adequate linearity over the ranges of 2–12 μg/mL for KET and 4–40 μg/mL for OLO.

**Table 1:** Regression parameters for determination of KET and OLO using the proposed methods.

Parameter	KET	OLO	
		^1^D_R_	DWM
Linearity range (μg/mL)	3–12	4–40	4–40
λ(nm)	323	234	243,291
r	0.9996	0.9997	0.9995
a	0.007	0.012	0.003
b	0.023	0.023	0.023
S_a_	0.007	0.006	0.005
S_b_	0.0003	0.0003	0.0003
S_y/x_	0.010	0.009	0.008
LOD (μg/mL)	1.06	0.92	0.97
LOQ (μg/mL)	3.21	2.78	2.96

r: correlation coefficient, a: intercept, b: slope, S_a_: standard deviation of intercept, S_b_: standard deviation of slope, S_y/x_: standard deviation of residuals, LOD: limit of detection (calculated), LOQ: limit of quantitation.

#### Limits of detection and quantitation

Estimation of limit of detection (LOD) and limit of quantitation (LOQ) for KET and OLO is necessary to ensure method sensitivity. Both are calculated (Table 1) using the equations: “*LOD* = *3.3S*_*a *_*/b*” and “*LOQ* = *10S*_*a *_*/b*” using the standard deviation of intercept (S_a_) and the slope of calibration curve (b). The calculated values for LOD [1.06 μg/mL for KET and “0.976 μg/mL, and 0.916 μg/mL” for OLO by ^1^D_R_ and DWM respectively] showed that both methods have adequate sensitivity for determination of KET and OLO in diluted solutions, bulk, and pharmaceutical dosage forms.

#### Accuracy

Assessment of accuracy is important to judge the trueness of analytical data. Three binary mixtures consisting of various concentration ratios of KET and OLO (three replicates) covering the linearity range of each were used to ensure the accuracy of the developed methods. The method’s accuracy was proved by the high value of % recovery (in the range of 98–102%) as shown in Table 2.

**Table 2:** Evaluation of accuracy for the determination of KET and OLO.

Drug (method)	Concentration taken (µg/mL)	Concentration found (µg/mL)			Mean concentration found* (µg/mL)	% Recovery	Mean %recovery ± SD
KET (Direct at 323 nm)	4.0	4.06	3.97	4.02	4.02	100.39	100.48 ± 0.14
	6.0	6.01	5.98	6.08	6.02	100.42	
	6.0	5.96	6.07	6.09	6.04	100.65	
OLO (^1^D_R_)	40.0	40.22	40.31	39.59	40.04	100.10	99.79 ± 0.71
	20.0	20.36	20.12	19.69	20.06	100.29	
	10.0	10.02	9.85	9.83	9.90	98.98	
OLO (DWM)	40.0	40.11	40.17	39.26	39.85	99.62	100.19 ± 0.59
	20.0	20.02	20.49	19.97	20.16	100.80	
	10.0	9.97	9.97	10.10	10.01	100.14	

*n = 3, S.D, standard deviation.

#### Precision

Evaluation of intra-day and inter-day precision was performed by performing analysis using the developed methods for various concentrations of KET and OLO covering the linearity range of each in three binary mixtures either in the same day or in three consecutive days. As shown in Table 3, the precision of the method is indicated by the small values of % relative standard deviation (% R.S.D).

**Table 3:** Evaluation of intra-day and inter-day precision for the determination of KET and OLO.

Drug (method)	Concentration taken (µg/mL)	Intra-day precision					Inter-day precision				
		Mean concentration found* (µg/mL)	SD	Mean %recovery	SD	%R.S.D	Mean concentration found* (µg/mL)	SD	Mean %recovery	SD	%R.S.D
KET (direct at 323 nm)	4.0	4.02	0.037	100.386	0.930	0.926	4.02	0.010	100.530	0.239	0.238
	6.0	6.02	0.044	100.416	0.737	0.734	6.04	0.027	100.616	0.449	0.446
	6.0	6.04	0.055	100.649	0.923	0.917	6.04	0.020	100.664	0.325	0.323
OLO (^1^D_R_)	40.0	40.04	0.322	100.096	0.805	0.804	40.02	0.013	100.058	0.033	0.033
	20.0	20.06	0.278	100.293	1.388	1.384	20.09	0.088	100.464	0.442	0.440
	10.0	9.90	0.083	98.985	0.834	0.842	10.01	0.090	100.063	0.905	0.904
OLO (DWM)	40.0	39.85	0.416	99.624	1.041	1.045	39.95	0.079	99.876	0.197	0.197
	20.0	20.16	0.236	100.800	1.182	1.173	20.07	0.094	100.373	0.468	0.466
	10.0	10.01	0.064	100.141	0.639	0.638	10.04	0.031	100.352	0.305	0.304

*n = 3, SD, standard deviation; % R.S.D, percentage relative standard deviation.

#### Specificity

**Figure 5:**
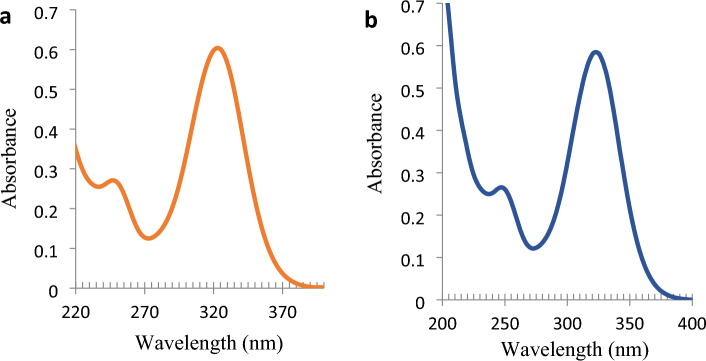
UV absorption spectra of standard binary mixture (**a**), and eye drop assay solution (**b**), both containing 2.0 µg/mL OLO and 8.0 µg/mL KET.

According to ICH-Q2R1 the method is confirmed to be specific as reliable results were obtained as mentioned in Table 4 indicating the absence of any expected matrix interferences. The UV spectrum of a binary mixture containing both drugs is found to be identical to that of tablet assay solution containing the same concentration of OLO and KET (Fig. 5).

**Table 4:** Determination of KET and OLO in Lab-prepared eye drop by the developed methods and the reported spectrophotometric method.

Drug (method)	Developed methods	Reported method ^18^	t-Test	F-test
	Mean % recovery* ± SD			
KET (direct at 323 nm)	99.63 ± 0.01	98.54 ± 0.01	1.07	1.14
OLO (^1^D_R_)	100.31 ± 0.01	100.40 ± 0.02	0.08	1.98
OLO (DWM)	100.90 ± 0.02	98.96 ± 0.02	1.27	1.85

*n = 3, Theoretical values for t‐test (0.05) is 2.77 and for F‐test (0.05) is 19.

### Application to lab-prepared eye drops

**Figure 6:**
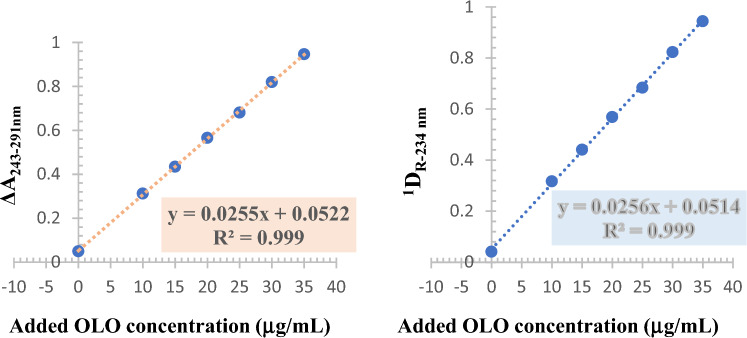
Multiple standard addition method for determination of OLO by DWM (left) and derivative ratio method (right).

A lab-prepared mixture simulating the dosage form was prepared as the eye drop preparation is not available in Egypt. The dosage form is composed of 0.4% w/v KET, 0.1% w/v OLO and, 0.01% w/v benzalkonium chloride. The presence of benzalkonium chloride did not interfere in the determination of the active ingredients as it is considered as a benzenoid poor absorbing chromophore that also present in a very low concentration. The challenge in this determination is how to determine OLO, a minor component with a poor UV absorptivity, with acceptable accuracy and precision in the presence of a major component with a supreme absorptivity (KET). Usually, for simultaneous analysis of similar drugs in such situation, either “sample enrichment of the minor component” or “dual measurements at two dilutions” approaches were employed. Both approaches were tried. The first approach ^5^ is based on spiking the assay solution, which is prepared in the same ratio of the dosage form, with a fixed amount of standard OLO to enrich the sample with OLO with the aim of increasing its concentration to reach the developed linearity range. Non-reliable results were obtained when using this approach, as the spiked amount of OLO exceeds that original amount of OLO in the eye drop assay solution. Furthermore, the second approach ^25^ failed to determine OLO concentrations with acceptable accuracy as measuring absorbance values for OLO from highly concentrated solutions increases the interference produced by the highly absorbing KET. A multiple standard addition method ^20^ was suggested to resolve this obstacle. The method is based on spiking the assay solution with increasing amounts of OLO standard solution as the expected total concentration of OLO lies within its linearity range. A standard addition curve representing the measured response versus the added amount of standard OLO is plotted and the amount of OLO in the assay solution was determined at the x-intercept of the regression line as shown in Fig. 6. Mean % recovery ± S.D for both drugs were determined (Table 4). The results were statistically compared to those obtained by a first derivative UV spectrophotometric reported method ^18^ and good agreement was attained.

### Greenness assessment

**Figure 7:**
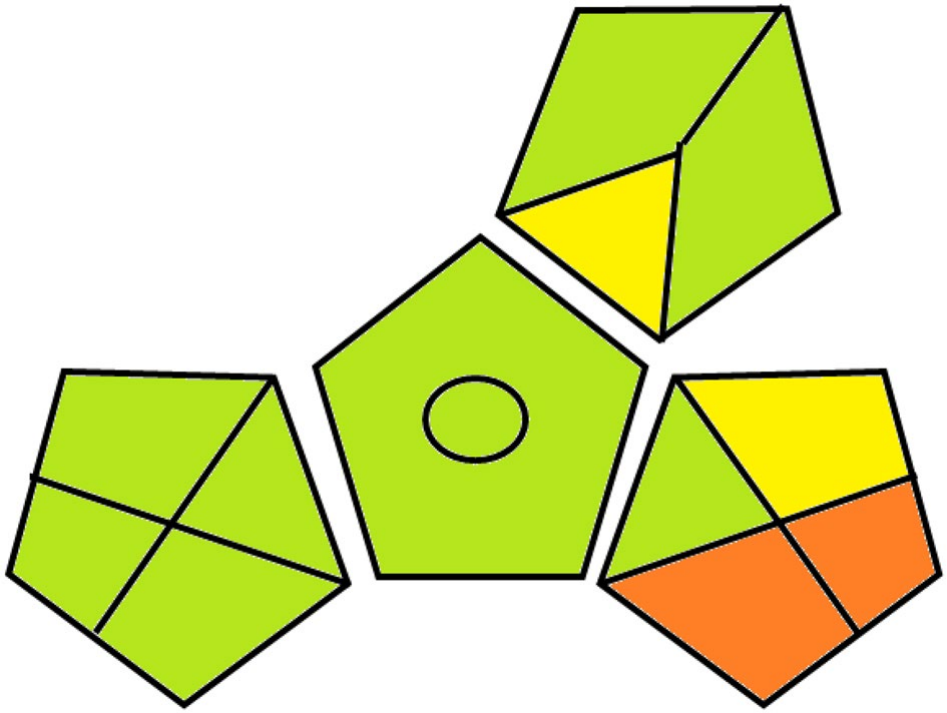
GAPI profile for the developed methods.

Analytical Eco-scale and GAPI were two procedures which were used to assess the developed methods’ greenness. The first procedure relied on calculating penalty points (PPs) from different factors such as waste, reagents, and instrumentation. A score was obtained by subtraction PPs from 100 to indicate greenness of the methods ^26^. Analytical Eco-scale tota; score of the developed methods was 92 which indicates excellent greenness (Table 5). In the second used assessment procedure, GAPI, the environmental impact of sample preparation, extraction, method type, reagents and waste have been represented in a form of five pentagrams using three colors: green, yellow or red ^27^. The almost green color in GAPI assessment (Fig. 7) indicates methods’ greenness.

**Table 5:** Eco-scale assessment of the developed methods.

Parameter	Value	Subtotal PPs*	Total PPs *
Reagents (water)			
Reagent amount	10–100 mL	2	0
Reagent hazard	None	0	
Instruments (UV/Vis spectrophotometer)			
Energy	< 0.1 kWh per sample	0	0
Occupational hazard		0	
Waste			
	(1–10 mL)	3	0
	No treatment	5	
Total penalty points (PPs)			8
Analytical Eco-scale total score **			92

*PPs means penalty points, Calculations of PPs were performed per sample, **Score = 100-total PPs.

## Conclusion

This work describes green simultaneous determination of KET and OLO by two validated and simple spectrophotometric methods. Determination of OLO relies on either measurement of the amplitude difference at two wavelengths in the zero-order spectrum or calculating the ratio spectra first derivative. Zero order UV spectrum has been used for directly quantification of KET at its wavelength of maximum absorption. The presented method’s sensitivity is comparable to the previously reported spectrophotometric methods and is suitable for quantification of KET and OLO in diluted solutions and in pharmaceutical dosage forms.

## Data Availability

All data generated or analyzed during this study are included in this article.
